# Validation of a High-Performance Liquid Chromatography with Photodiode Array Detection Method for the Separation and Quantification of Antioxidant and Skin Anti-Aging Flavonoids from *Nelumbo nucifera* Gaertn. Stamen Extract

**DOI:** 10.3390/molecules27031102

**Published:** 2022-02-07

**Authors:** Duangjai Tungmunnithum, Samantha Drouet, Christophe Hano

**Affiliations:** 1 Department of Pharmaceutical Botany, Faculty of Pharmacy, Mahidol University, Bangkok 10400, Thailand; 2 Laboratoire de Biologie des Ligneux et des Grandes Cultures, INRAE USC1328, Campus Eure et Loir, Orleans University, 28000 Chartres, France; samantha.drouet@univ-orleans.fr; 3 Le Studium Institue for Advanced Studies, 1 Rue Dupanloup, 45000 Orléans, France

**Keywords:** green extraction, lotus plant, antioxidant flavonoids, skin-aging enzyme inhibition, cosmetic application, HPLC column (core shell column)

## Abstract

*Nelumbo nucifera* Gaertn., or the so-called sacred lotus, is a useful aquatic plant in the Nelumbonaceae family that has long been used to prepare teas, traditional medicines as well as foods. Many studies reported on the phytochemicals and biological activities of its leaves and seeds. However, to date, only few studies were conducted on its stamen, which is the most important ingredient for herbal medicines, teas and other phytopharmaceutical products. Thus, this present study focuses on the following: (1) the application of high-performance liquid chromatography with photodiode array detection for a validated separation and quantification of flavonoids from stamen; (2) the *Nelumbo nucifera* stamen’s in vitro and in cellulo antioxidant activities; as well as (3) its potential regarding the inhibition of skin aging enzymes for cosmetic applications. The optimal separation of the main flavonoids from the stamen ethanolic extract was effectively achieved using a core-shell column. The results indicated that stamen ethanolic extract has higher concentration of in vitro and in cellulo antioxidant flavonoids than other floral components. Stamen ethanolic extract showed the highest protective effect against reactive oxygen/nitrogen species formation, as confirmed by cellular antioxidant assay using a yeast model. The evaluation of potential skin anti-aging action showed that the stamen extract has higher potential to inhibit tyrosinase and collagenase compared with its whole flower. These current findings are the first report to suggest the possibility to employ *N. nucifera* stamen ethanolic extract as a tyrosinase and collagenase inhibitor in cosmetic applications, as well as the utility of the current separation method.

## 1. Introduction

**Figure 1:**
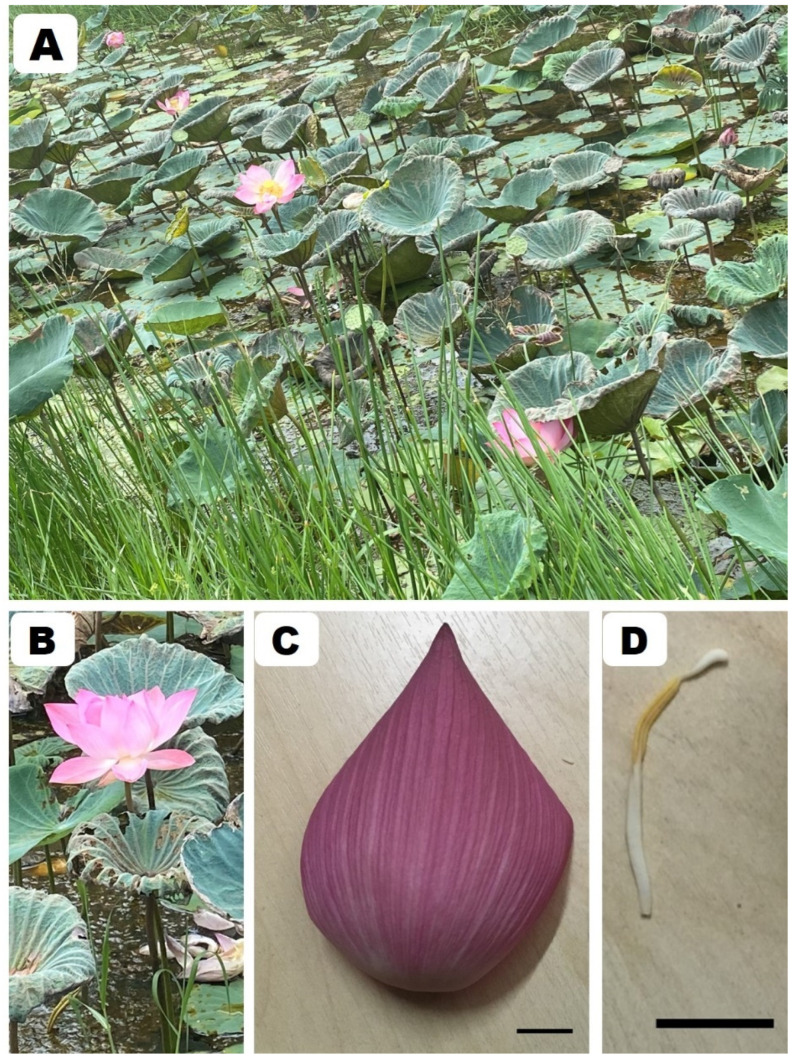
*Nelumbo nucifera* Gaertn.: (**A**) habitat; (**B**) the whole flower; (**C**) petal; (**D**) stamen; bar scale = 1 cm. Pictures by Duangjai Tungmunnithum.

Sacred lotus is an aquatic flowering plant (Figure 1), which is a species member of the family Nelumbonaceae.

Lotus is a medical plant distributed in Asian regions such as Thailand, Japan, China, India, Sri Lanka and so forth [1,2,3,4,5,6,7,8]. This plant has long been used in foods, herbal teas and as an ingredient for traditional medicines in many Asian countries [1,3,4,6,7]. Its flower and stamen are the most important parts for traditional medicine and herbal tea preparation, offering health benefits such as improved blood circulation and boosted immune systems [6,7,9,10]. This lotus plant is recognized using various vernacular names depending on its distribution, e.g., บัวหลวง (Bau Luang) in Thailand, 莲 (Lian) in China and ロータス (Ro Tasu) in Japan. Furthermore, this plant is also called by its common name such as sacred lotus, water lily or Indian lotus [1,6,9,10,11]. However, the legitimate name or the scientific name of this plant species is known as *Nelumbo nucifera* Gaertn. 

As this lotus plant is widely used in both foods and herbal medicines, there are a large number of studies on its biological activities [8,9,10,11,12,13]. Most of the previous research studies focus on the leaves of *N. nucifera*; for example, Lin and his team [3] isolated flavonoid phytochemical compounds from a leaf extract and reported on their antioxidant activity. In 2015, Zhu et al. [5] discovered four new flavonoid compounds from *N. nucifera* leaf extract and reported on their strong antioxidant potential. Besides the antioxidant activity, the anti-inflammatory effect [14], anti-obesity effects [15], the potential against ultraviolet B-induced phototoxicity in both in vitro and in vivo models [16] as well as the ability to inhibit cell proliferation of breast cancer in vitro and in vivo were also reported for the leaves of this lotus species. The antioxidant potential from methanolic extract of *N. nucifera* stamen was reported in 2003 [9]. In addition, antioxidant activity was reported for the 50% (*v*/*v*) hydroalcoholic extract from the seed of this medicinal plant [2]. Chen et al. [17] also reported on the antioxidant potential of flavonoids from *N. nucifera* epicarp, whereas Zhu and his team in 2017 [18] as well as Jiang and his team in 2018 [19] conducted their studies on the embryo of *N. nucifera*. These two research teams indicated a similar trend regarding the antioxidant potential of flavonoid glycosides and other phytochemical compounds from the embryo of this plant. Nowadays, it is clearly seen that there are few studies investigating the biological activities of the flower and stamen of *N. nucifera*, which are the most necessary parts for herbal teas and traditional medicines. A representative high-performance liquid chromatography (HPLC) reference pattern was recently produced for *N. nucifera* stamen [7]. Seven flavonoid glycosides were derived from *N. nucifera* stamen extract: three were derived from kaempferol (Kae: Kae-3-*O*-robinobioside, Kae-3-*O*-glucoside, and Kae-3-*O*-glucuronic acid), two were derived from quercetin (Que: Quer-3-*O*-Rut and Quer-3-*O*-glucuronic acid), one was derived from myricetin (Myr: Myr-3-*O*-glucoside), and one was derived from isorhamnetin (Iso: Iso-3-*O*-glucoside) [7]. These flavonoids could be employed as quality control indicators for potential medical and cosmetic uses of *N. nucifera* stamen extract. Validation of analytical methods is required for this purpose. In particular, for the separation and quantification of flavonoids and their glycosides, many analytical methods have been developed, with high-performance liquid chromatography (HPLC) with photodiode array (PDA) detection being the most extensively used [20,21]. 

In this paper, we describe a validated HPLC-PDA analytical approach using a core-shell column for the separation and quantification of flavonoids from *N. nucifera* stamen ethanolic extract with high resolution and precision for cosmetic applications. The method was validated in terms of the resolution, linearity, precision, accuracy, limit of detection (LOD), and limit of quantification (LOQ) of each flavonoid. In vitro and in cellulo antioxidant activity, as well as the inhibition of skin aging enzymes, were used to assess the cosmetic potential of the extracts.

## 2. Results and Discussion

### 2.1. Preliminary Screening of N. nucifera Flower Parts

The total flavonoid content (TFC) and the DPPH radical scavenging activities of different flower parts of *N. nucifera* were assessed using t ultrasound-assisted extraction followed by flavonoid enrichment using DAX-8 macroporous resin (USAE-MPR), which was previously described for the extraction of flavonoids from another lotus species, *Nymphea lotus* [22].

The results in Table 1 indicate that *N. nucifera* stamen have a higher concentration of antioxidant flavonoids than other floral components. This result is consistent with previous reports on *N. nucifera* [9] and other lotus species [22]. The stamen is the pollen-bearing reproductive organ of flowers. Flavonoids found in pollen grains have been demonstrated to increase plant development and influence sexual reproduction owing to their antioxidant activity, which reduces the amount of reactive oxygen species (ROS) [23].

### 2.2. Separation and Quantification Validation of Flavonoids from N. nucifera Stamen Extract by High-Performance Liquid Chromatography with Photodiode Array Detection

**Figure 2:**
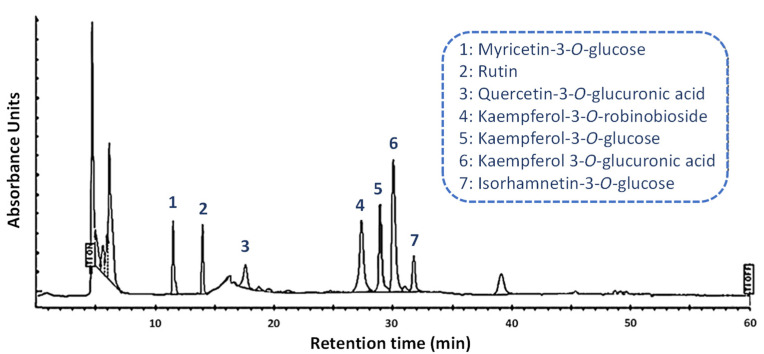
Reversed-phase high-performance liquid chromatography (HPLC) with photodiode array (PDA) detector showing the separation of the flavonoids from *N. nucifera* stamen ethanolic extract (here recorded at 320 nm).

An optimal separation of the main flavonoids from *N. nucifera* stamen ethanolic extract was achieved using a C18 core-shell column with iso-butyl side chains (Kinetex C18, 5 µm core-shell technology, Phenomenex) with photodiode array detection, and a mobile phase consisting of a mixture of methanol and HPLC-grade water, both acidified with 0.05% (*v*/*v*) formic acid (Figure 2). 

Here, the use of a core-shell column improved the resolution when compared to our results obtained with a fully porous C18 column [22]. Core-shell columns are thought to provide a higher efficiency, better peak symmetry, and greater resolution for the analysis of different flavonoids as compared to the most common monolithic columns [20,24]. Furthermore, the core-shell technology is sufficiently versatile to make the current protocol more easily adaptable to other techniques such as UPLC, as previously demonstrated [25].

Each compound was separated with high repeatability, as indicated by the relative standard deviations in their retention time values (Table 2). The effective separation of the seven mains flavonoids from *N. nucifera* stamen ethanolic extract was observed with a correct resolution of the method, as indicated by the Rs values (Table 2). Indeed, to meet the baseline-separation requirements, R_s_, the value representing the degree of separation between adjacent components must be higher than 1.5 [20,21], as observed in the present case. The symmetry factors were also evaluated, and with values ranging from 0.97 to 1.08, the symmetry of the peaks of all studied flavonoids was adequate (Table 2). The retention times had RSDs of less than 0.95%. The six-point calibration curves exhibited good linearities, with correlation values over 0.998, implying that the responses to the external standards were linearly suitable over the studied 0.5–100.00 µg/mL range (Table 2). The terms LOD and LOQ are used to assess a method’s ability to detect and quantify compounds at low concentrations [20,21]. The response standard deviation and slope of the calibration curves were used to determine the LODs and LOQs, which revealed that the current approach is properly sensitive for quantifying flavonoids from *N. nucifera* stamen ethanolic extract (Table 2).

The method was then verified according to the criteria of the Association of Analytical Communities (AOAC) to ensure accuracy and reproducibility in the quantification [26]. Table 3 summarizes the validation results, including the Horwitz ratio, the accuracy as well as both the intra- and inter-day precision.

For this purpose, the concentrations of the flavonoids were quantitively determined (n = 5) in *N. nucifera* stamen ethanolic extract. The repeatability precision of the separation method was assessed in terms of both the percentage of relative standard deviation (RSD), which was as low as 4.16%, and the Horwitz ratio (HorRat, an index of method performance with respect to precision), which was as low as 1.68 [27]. The accuracy was determined with three spiked concentrations of 25, 50 and 100 µg/mL and expressed as mean recovery values. The degree to which the measured value was near to the actual value is referred to as accuracy. All the recovery values ranging from 98.49 to 102.40% (i.e., close to 100% recovery values) suggested that the current separation approach is accurately quantitative (Table 3). The intra-day repeatability and inter-day reproducibility on five distinct days were used to determine precision, which was represented as percentage and RSD. The intra-day repeatability RSDs were found to be 1.68–3.18% when tested six times in a single day, whereas the inter-day reproductivity RSDs on five distinct days were found to be 1.99–4.40% (Table 3). The RSD values being lower than 5% demonstrated that the established analytical method was precise in the quantification of the seven flavonoids tested in this study [26]. This opens the door to further research into the natural variability of these compounds’ concentrations in different wild populations of *N. nucifera*, or plants produced under different conditions, or the authentication of *N. nucifera* stamen ethanolic extracts, which are crucial information for possible cosmetic applications [22,28]. 

### 2.3. Evaluation of Potential Cosmetic Activities of N. nucifera Extracts 

**Figure 3:**
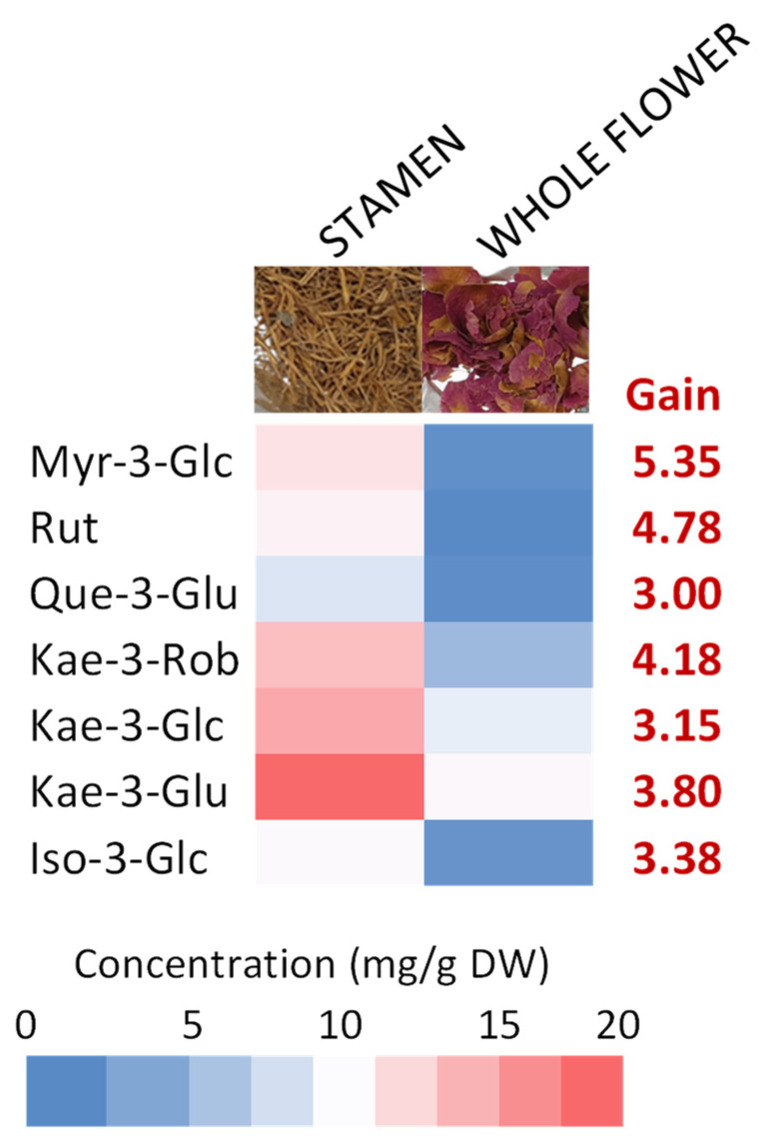
Quantification of the major flavonoids from *N. nucifera* stamen ethanolic extract compared to the whole flower ethanolic extract. The means and standard deviations (n = 3) are provided in Table S1. The gain is the ratio, for each analyte, of their concentrations in stamen extract divided by their concentrations in whole flower extract. Myr: myricetin; Myr-3-*O*-Glc: Myr-3-*O*-glucoside; Que: quercetin; rutin: Quer-3-*O*-Rut (with rut: rutinoside); Quer-3-*O*-Glu: Quer-3-O-glucuronic acid; Kae: kaempferol; Kae-3-*O*-Glc: Kae-3-O-glucoside; Kae-3-O-Rob: Kae-3-*O*-robinobioside; Kae-3-*O*-Glu: Kae-3-*O*-glucuronic acid; Iso: isorhamnetin; Iso-3-*O*-Glc: Iso-3-*O*-glucoside.

The developed HPLC method was then used to analyze and quantify the seven flavonoids in *N. nucifera* stamen and whole flower ethanolic extract (Figure 3; Table S1 in Supplementary Materials).

The highest concentrations were measured in *N. nucifera* stamen ethanolic extract; the individual flavonoid concentrations ranged from 3.21 (Quer-3-O-Glu) to 16.05 (Kae-3-O-glucuronic acid) mg/g DW. The gains obtained with stamen as the starting material varied from 3.00 (Quer-3-O-Glu) for the lowest gain to 5.35 (Myr-3-O-Glc) for the highest gain. Only few studies dealt with the flavonoids from *N. nucifera* stamen extracts [6,8,9,22,29]. The presence of the three kaempferol glycosides was previously reported but not quantified [9]. Temviriyanukul et al. [8] reported concentrations for aglycones produced by acidic hydrolysis that are consistent with those obtained in the present study. The present quantitative results demonstrated that the *N. nucifera* stamen may be a richer starting material in flavonoids compared to the whole flowers, indicating a better potential for cosmetic uses. Different activities (in vitro and cellular antioxidant and skin anti-aging enzyme inhibition) were determined for both extracts to confirm this (Table 4).

The results of each of the in vitro antioxidant assays presented in Table 4 are expressed in µmol of Trolox C-equivalent antioxidant capacity (TEAC). The in vitro antioxidant assays relied on both electron transfer (ET) (for FRAP and CUPRAC assays) and hydrogen atom transfer (ABTS and ORAC assays), but the DPPH assay is often regarded as a mixed assay (i.e., HAT but also ET) [30]. When comparing the different antioxidant activities, the ET-based assays had the highest antioxidant activity, indicating that the HAT-based mechanism is more important than the ET-based mechanism for the antioxidant action of these ethanolic extracts. A stronger correlation between the ET-based mechanism and some flavonoids has been previously reported [31,32]. Compared to the whole flower extract, the stamen extract was found to have stronger in vitro antioxidant capacity, which might be due to its higher flavonoid concentration. This result is consistent with previous results [8], in particular those of Jung et al. [9], whose were the first to demonstrate that flavonoids from *N. nucifera* stamen extract, particularly Kaempferol glycosides, have radical scavenging potential. This result was here further confirmed using a cellular antioxidant assay (CAA) performed on yeast cells subjected to UV-induced oxidative stress. Stamen ethanolic extract showed the highest protective effect against ROS and RNS formation (Table 4). This result is consistent with previously reported antioxidant capacities of stamen yeast cells, which have long been employed to test the antioxidant potential of different extracts or compounds [33,34,35]. It is a solid eukaryotic model with well-known mechanisms involved in oxidative stress protection and/or adaptation that may easily be adapted to humans due to molecular processes that are highly conserved within eukaryotic cells [36]. As a direct result of redox cellular imbalances, the generation of ROS and RNS rises with age, stress or pollution, and has been connected to aging processes and may contribute to the development of a range of degenerative illnesses [37]. Thus, the current findings supported the potential preventive action of various *N. nucifera* extracts against chronic degenerative diseases [6,8], as well as the utility of the current separation method and the possibility of using the stamen extract as a nutraceutical or cosmeceutical.

The next step consisted in the evaluation of the potential skin anti-aging action of the extracts determined as their in vitro abilities to inhibit elastase, hyaluronidase, collagenase (Matrix Metalloproteinase type 1 (MMP1)) and tyrosinase activities. The ability of *N. nucifera* stamen ethanolic extract to inhibit tyrosinase and collagenase was found to be higher than that of whole flower ethanolic extract. On the contrary, the inhibitory effects of elastase and collagenase were less pronounced for both extracts (Table 4). The extracellular matrix components in the dermis are degraded by enzymes such as elastase, hyaluronidase, and collagenase, leading in a loss of skin tone, the creation of deep wrinkles and reduced resilience [38,39,40]. Dysfunctions of the tyrosinase enzyme progress with age and can result in malignant melanoma, as well as pigmentary abnormalities such as freckles or melisma [41]. As a result, natural compounds and/or extracts that can inhibit these enzyme activities or processes have received a lot of interest in the cosmetics industry. Several flavonoids have been previously reported as tyrosinase and/or collagenase inhibitors [42,43]. The stamen extract, which was richer in flavonoids, had stronger tyrosinase and collagenase inhibition capacity than the whole flower extract, which was in line with these observations. Our results are the first to suggest the possibility of using *N. nucifera* stamen extract as a tyrosinase and collagenase inhibitor for cosmetic applications.

## 3. Materials and Methods

### 3.1. Plant Materials 

The *N. nucifera* living specimens were collected from their natural aquatic habitat in the Nonthaburi Province of Thailand. The collected samples were identified at the species level using the taxonomic key as well as the species descriptions of the existing Floras [1,44], and then compared with the herbarium specimens kept in Forest Herbarium (BKF), Bangkok, Thailand, by Prof. Kasin Suvatabandhu from the Herbarium, Chulalongkorn University (BCU). The herbarium abbreviations are used following Thiers et al. [45]. The whole flowers, perianth and stamen were then air-dried, and prepared following World Health Organization recommendations [46]. 

### 3.2. Chemicals

All of the reagents and solvents that were used for extraction and HPLC analysis were of analytical grade or the highest available purity (Thermo Fischer Scientific, Illkirch, France). The deionized water was purified using the Milli-Q water-purification system (Merck Millipore Fontenay sous Bois, Paris, France). In addition, all the solutions that were prepared for HPLC analysis were filtered through 0.45 µm nylon syringe membranes prior to use. The standards were purchased from Extrasynthese (Genay, France). 

### 3.3. Extraction

The dried stamen or whole flower samples (100 mg) were placed in 5 mL quartz tubes equipped with a vapor condenser, and then extracted by means of ultrasound-assisted extraction in 1 mL 90% (*v*/*v*) aqEtOH in the USC1200TH ultrasonic bath (Prolabo, Fontenay-sous-Bois, France), following optimized extraction conditions: 30 kHz frequency for 45 min at 45 °C [22]. The extract was centrifuged for 15 min at 5000× *g* (Heraeus Biofuge Stratos, Thermo Scientific, Illkirch, France), and then the obtained supernatant was filtered through 0.45 μm nylon syringe membranes (Merck Millipore, Saint-Quentin Fallavier, France). The flavonoid enrichment was obtained through the additional DAX-8 (Merck Millipore, Saint-Quentin Fallavier, France) macroporous resin purification step as described in a previous study [22].

### 3.4. HPLC Analysis

The high-performance liquid chromatography system, consisting of an autosampler, a Varian (Les Ulis, France) Prostar 230 pump and a Varian Prostar 335 photodiode array detector, was used; this was controlled using Galaxie software (Varian v1.9.3.2). The separation was performed at 35 °C using a C18 core-shell column with iso-butyl side chains with TMS endcapping (Kinetex 5 µm XB-C18, 100 Å, LC Column 150 × 4.6 mm, core-shell silica, Phenomenex Le Pecq France). The mobile phase composed of a methanol (solvent A) and HPLC-grade water (solvent B) mixture, both being acidified with 0.05% formic acid. A linear gradient was applied for the mobile phase variation from a 5:95 (*v*/*v*) to a 100:0 (*v*/*v*) mixture of solvents A and B, respectively; the flow rate was 1.30 mL/min and the injection volume was 3 µL. The maximum back pressure was 110 bar. Detection was conducted using PDA, and the quantification was performed at 320 nm (the maximum absorbance wavelength of the studied flavonoids). Flavonoid compounds were identified by means of comparison with the authentic standards (Extrasynthese, Genay, France). The limits of detection (LOD) and limits of quantification (LOQ) were investigated based on the signal-to-noise ratios (S:N) of 3:1 and 10:1, respectively, following the previous study [20].

### 3.5. Method Validation

The separation method was validated following a previously described approach [20]. The linear correlations between peak area and standard concentrations were found to be high, in the range between 0.5 and 100 µg/mL. The linear equations were gained with R^2^ values for a six-point calibration graph > 0.99, and the slopes of the 5 replicates of the calibration graph covering the analytical range for each standard varied by less than 1% in terms of RSD over a period of 4 weeks. The repeatability precision was then evaluated using six injections of the same sample, which were performed on the same day. The accuracy was determined by the calculation of the recovery, the RSD and the average recovery using spiked concentration additions at 3 different levels: 25, 50 and 100 µg/mL.

### 3.6. Determination of Total Flavonoid Content

Total flavonoid content (TFC) was determined using the aluminum trichloride (AlCl_3_, Sigma Aldrich) colorimetric method, as previously reported [22], with minor modifications. In brief, 10 μL of the sample was mixed with 10 μL of potassium acetate (1 M), 10 μL of AlCl_3_ (10%, *w*/*v*) and 170 µL of double distilled water. After that, the mixture was incubated at room temperature (25 ± 2 °C) for 30 min, and then the absorbance was measured at 415 nm using a UV–visible spectrophotometer (BioTek ELX800 Absorbance Microplate Reader, BioTek Instruments). The quercetin standard (Sigma Aldrich) was used for the calibration curve, and the TFC were expressed in milligrams of quercetin equivalent per gram.

Total flavonoid production (mg·L^−1^) = DW (g·L^−1^) × TFC (mg·g^−1^).

### 3.7. In vitro Antioxidant Assays

#### 3.7.1. Antioxidant DPPH Assay

The 20 μL/sample extract was used in combination with 180 μL of DPPH (2,2-Diphenyl-1-picrylhydrazyl) reagent, and the absorbance was recorded at 517 nm using a BioTek ELX800 Absorbance Microplate Reader (BioTek Instruments, Colmar, France). The assays were made in triplicate and the results were expressed in terms of Trolox C-equivalent antioxidant capacity [30].

#### 3.7.2. Antioxidant ORAC Assay

The oxygen radical absorbance capacity assay (ORAC) was performed as suggested in a previous study [47]. Briefly, 10 μL of extracted sample was mixed with 190 μL of 0.96 µM fluorescein in 75 mM phosphate buffer (pH 7.4), and then incubated at 37 °C for 20 min. After that, 20 µL of 119.4 mM 2,2′-azobis-amidinopropane (ABAP) was added; the fluorescence intensity was recorded every 5 min for 2.5 h at 37 °C with the use of a fluorescence spectrophotometer (BioRad, Marnes-la-Coquette, France) that was set with the excitation at 485 nm and the emission at 535 nm. The assays were made in triplicate. The results of the ORAC assay were expressed in terms of the Trolox C-equivalent antioxidant capacity.

#### 3.7.3. Antioxidant ABTS Assay

The 2,2-azinobis (3-ethylbenzothiazoline-6-sulphonic acid (ABTS) assay was accomplished using the method developed by Velioglu et al. [48]. Briefly, ABTS solution was made from a mixture containing an equal proportion of ABTS salt (7 mM) and potassium persulphate (2.45 mM), and the mixture was kept in the dark for 16 hours. The absorbance of the solution was measured at 734 nm, and then adjusted to 0.700 and mixed with the extract. After that, the mixture was kept in the dark at 25 ± 1 °C for 15 min and the absorbance was recorded at 734 nm using a BioTek ELX800 Absorbance Microplate Reader (BioTek Instruments, Colmar, France). The assays were made in triplicate and the results were expressed in terms of Trolox C-equivalent antioxidant capacity.

#### 3.7.4. Antioxidant FRAP Assay

The ferric-reducing antioxidant power (FRAP) assay was modified from the method of Benzie and Strain [49]. Briefly, 10 μL of the extract was mixed with 190 μL of FRAP solution, which was composed of 20 mM FeCl3, 10 mM TPTZ, 6H_2_O and 300 mM acetate buffer (pH 3.6) at a ratio of 1:1:10 (*v*/*v*/*v*). Then, the reaction mixtures were incubated at 25 ± 1 °C for 15 min. The absorbance of the reaction mixture was determined at 630 nm using the BioTek ELX800 Absorbance Microplate Reader (BioTek Instruments). The assays were made in triplicate and the results were expressed in terms of Trolox C-equivalent antioxidant capacity.

#### 3.7.5. Antioxidant CUPRAC Assay

The cupric ion-reducing antioxidant capacity (CUPRAC) assay was conducted using a modified version of the method of Apak et al. [50]. In brief, 10 μL of extract and 190 μL of CUPRAC reaction solution, which contained 7.5 mM neocuproine, 10 mM Cu(II) and 1 M acetate buffer (pH 7) at a ratio of 1:1:1 (*v*/*v*/*v*), were mixed. After that, the reaction mixtures were incubated at 25 ± 1 °C for 15 min. The absorbance was recorded at 450 nm using the BioTek ELX800 Absorbance Microplate Reader (BioTek Instruments). The assays were made in triplicate and the results were expressed in terms of Trolox C-equivalent antioxidant capacity.

### 3.8. Cellular Antioxidant Assay

Yeast strain DBY746 (MAT leu2-3,112 his31 trp1-289a ura3-52 GAI+; ATCC 204660) culture was started with frozen stock plated onto the yeast extract peptone dextrose (YPD) medium (Sigma-Aldrich, Saint-Quentin Fallavier, France). The extract, at a final concentration of 1 mg/mL, was dissolved in cell culture-grade dimethyl sulfoxide (DMSO; Sigma-Aldrich, Saint-Quentin Fallavier, France), and then applied at a final DMSO concentration of 0.1% (*v*/*v*). The control yeast was inoculated with the same DMSO concentration. Resveratrol was used as a positive control at a final concentration of 10 µM. The impact on yeast survival was asserted as previously described [33].

Firstly, the yeast cells were treated under the same conditions as described above. Yeast cells were irradiated with a UV dose of 106.5 J/m2 UV-C (254 nm) under the Vilber VL-6.C filtered lamp (Thermo Fisher Scientific, Villebon-sur-Yvette, France), and then incubated at 28 °C with orbital shaking at 120 rpm in the dark in the complete 2.0% (*w*/*v*) glucose YPD medium (Sigma Aldrich, Saint-Quentin Fallavier, France), as previously described [33]. Similar conditions were used to grow non-irradiated cells. Hour 0 of the oxidative stress experiment was considered as the beginning of irradiation.

Dihydrorhodamine-123 (DHR-123) fluorescent dye (Sigma-Aldrich, Saint-Quentin Fallavier, France) was used to evaluate the quantity of reactive oxygen and nitrogen species (ROS and RNS). Approximately 10^8^ yeast cells were washed 2 times in PBS, resuspended in PBS containing 0.4 M DHR-123, and then incubated in the dark at 28 °C for 10 min in the presence of the extract, 10 µM resveratrol (positive control) or DMSO (control cells). The fluorescence signal (ex = 505 nm, em = 535 nm) was measured using the VersaFluor Fluorimeter after washing twice with PBS (Biorad, Marnes-la-Coquette, France).

### 3.9. Enzyme Inhibitions 

#### 3.9.1. Collagenase Assay

Collagenase clostridium histolyticum (Sigma Aldrich) was used for this experiment, and its activity was determined with the aid of a spectrophotometer using N-[3-(2-furyl)acryloyl]-Leu-Gly-Pro-Ala (FALGPA; Sigma Aldrich) as the substrate in accordance with the protocol of Wittenauer et al. [51]. The decrease in the absorbance of the FALGPA was followed for 20 min at 335 nm using a microplate reader (BioTek ELX800; BioTek Instruments, Colmar, France). The measurements were conducted in triplicate, and the anti-collagenase activity was revealed as the percent inhibition relative to the control (extraction solvent) for every extract sample. 1,10-Phenantroline (100 µM) was used as the specific inhibitor of collagenase.

#### 3.9.2. Elastase Assay

The elastase assay was performed using porcine pancreatic elastase (Sigma Aldrich) and its activity was investigated with a spectrophotometer using N-Succ-Ala-Ala-Ala-p-nitroanilide (AAAVPN; Sigma Aldrich) as the substrate and following *p*-nitroaniline’s release at 410 nm using a microplate reader (BioTek ELX800; BioTek Instruments) modified based on the method described by Wittenauer et al. [51]. The measurements were conducted in triplicate, and the anti-elastase activity was revealed as the percent of inhibition relative to the control (extraction solvent) for every extract sample. Oleanolic acid (10 µM) was used as the specific inhibitor of elastase.

#### 3.9.3. Hyaluronidase Assay

The hyaluronidase inhibitory assay was performed following the suggested method of Kolakul et al. [52] using 1.5 units of hyaluronidase (Sigma Aldrich) as well as hyaluronic acid solution (0.03% (*w*/*v*)) as the substrate. The precipitation of the undigested form of hyaluronic acid occurred with acid albumin solution (0.1% (*w*/*v*) BSA). Then, absorbance was measured at 600 nm using a microplate reader (BioTek ELX800; BioTek Instruments, Colmar, France). Hyaluronidase’s inhibitory effect was expressed as the percent of inhibition relative to the control (extraction solvent) for every extract sample. Oleanolic acid (10 µM) was used as a specific inhibitor of hyaluronidase.

#### 3.9.4. Tyrosinase Assay

The tyrosinase assay was conducted following the method described Chai et al. [53]. Briefly, l-DOPA (5 mM; Sigma Aldrich) was used as the diphenolase substrate, and then mixed in sodium phosphate buffer (50 mM, pH 6.8) with 10 µL of the extract. Lastly, 0.2 mg/mL of mushroom tyrosinase solution (Sigma Aldrich) was added to this mixture, in order to reach the final volume of 200 µL. Control tests, using an equal amount of extraction solvent to replace the extract sample, were routinely carried out. The reaction processes were detected using a microplate reader (BioTek ELX800; BioTek Instruments) at a wavelength of 475 nm. Tyrosinase’s inhibitory effect was expressed as the percent of inhibition relative to the control. Kojic acid (10 µM) was used as the specific inhibitor of tyrosinase.

### 3.10. Statistical Analysis 

The statistical analysis was performed using the XLSTAT 2019 suite (Addinsoft, Paris, France). The data contained at least 3 independent replicates, and the analyzed data were presented as means and standard deviations. The Student’s t-test was employed for statistical comparative analysis. Significant differences at *p* < 0.05, 0.01 and 0.001 were represented by *, ** and ***. Different letters were used to indicate the significant differences at *p* < 0.05.

## 4. Conclusions

The findings of the current study revealed that the validated HPLC separation, conducted using a C18 core-shell column with iso-butyl side chains with PDA detection, is a suitable approach to separate with high resolution and to precisely quantify the main flavonoids’ glycosides in *N. nucifera* stamen ethanolic extract. The present stamen ethanolic extract obtained following ultrasound-assisted extraction and macroporous resin enrichment was richer in flavonoids, higher in vitro antioxidant capacity and showed stronger skin anti-aging action (tyrosinase and collagenase inhibition) compared to the whole flower ethanolic extract. Considering that its cytotoxicity in humans is less concerning due to the fact that this raw plant material has been consumed as a food, tea and herbal drug since ancient times, the present results provide fundamental and clear evidence that the stamen of *N. nucifera* is a potential raw material that can be effectively employed in the development of cosmetic as well as other phytopharmaceutical products. 

## Figures and Tables

**Table 1:** Total flavonoid content (TFC, in mg/g dry weight (DW)) and DPPH radical scavenging activity (in µmol Trolox-equivalent antioxidant activity (TEAC)) of different *N. nucifera* flower parts obtained after ultrasound-assisted extraction followed by flavonoid enrichment using DAX-8 macroporous resin (USAE-MPR).

	TFC (mg/g DW)	DPPH Radical Scavenging Activity (µmol TEAC)
Whole flower	40.08 ± 1.94 ^b^	89.91 ± 2.64 ^b^
Stamen	68.11 ± 3.53 ^a^	183.69 ± 4.84 ^a^
Petal	38.67 ± 0.70 ^b^	83.19 ± 1.33 ^b^

Data are expressed as means ± standard deviations of *n* = 3 independent measurements. Different letters represent significant differences between the various extracts (*p* < 0.05).

**Table 2:** Calibration function parameters for the main flavonoids from *N. nucifera* stamen ethanolic extract using UV detection.

Flavonoid	Retention Time (t_R_)				Calibration Curve			LOD (µg/mL)	LOQ (µg/mL)
	Min	RSD (%)	R_s_	Sym Fact	Slope	Intercept	R^2^		
Myr-3-*O*-Glc	11.63	0.95	5.92	1.03	1909.9	474.6	0.999	0.08	0.25
Rutin	14.12	0.64	3.34	0.99	1961.3	308.5	0.999	0.05	0.16
Quer-3-*O*-Glu	17.44	0.69	1.68	1.16	2438.9	368.9	0.998	0.05	0.15
Kae-3-*O*-Glu	27.28	0.54	2.83	1.05	2880.6	867.6	0.998	0.10	0.30
Kae-3-*O*-Rob	28.89	0.65	3.21	0.97	3113.6	208.7	0.999	0.02	0.07
Kae-3-*O*-Glc	30.48	0.92	1.65	0.98	3038.6	345.5	0.999	0.04	0.11
Iso-3-*O*-Glc	31.96	0.53	1.67	1.08	1959.1	794.2	0.998	0.13	0.41

Myr: myricetin; Myr-3-*O*-Glc: Myr-3-*O*-glucoside; Que: quercetin; rutin: Quer-3-*O*-Rut (with rut: rutinoside); Quer-3-*O*-Glu: Quer-3-O-glucuronic acid; Kae: kaempferol; Kae-3-*O*-Glc: Kae-3-*O*-glucoside; Kae-3-*O*-Rob: Kae-3-*O*-robinobioside; Kae-3-*O*-Glu: Kae-3-*O*-glucuronic acid; Iso: isorhamnetin; Iso-3-*O*-Glc: Iso-3-*O*-glucoside; RSD: relative standard deviation; R_s_: resolution value; Sym Fact: symmetry factor; R^2^: correlation coefficient; LOD: limit of detection; LOQ: limit of quantification.

**Table 3:** Quantification and validation parameters for the simultaneous analysis of the main flavonoids from *N. nucifera* stamen ethanolic extract.

Flavonoid	Concentration	RSD (%)	HortRat	Accuracy		Intra-Day Precision		Inter-Day Precision	
	(mg/g DW)			Recovery (%)	RSD	%	RSD	%	RSD
Myr-3-*O*-Glc	5.94 ± 0.23	3.87	1.48	98.49	3.53	97.98	2.02	96.80	3.20
Rutin	4.68 ± 0.14	2.99	1.19	102.40	2.34	97.44	2.56	97.01	2.99
Quer-3-*O*-Glu	3.21 ± 0.09	2.80	1.18	101.15	2.14	96.88	3.12	96.26	3.74
Kae-3-*O*-Glu	8.99 ± 0.21	2.34	0.84	102.18	2.13	97.44	2.56	96.89	3.11
Kae-3-*O*-Rob	10.81 ± 0.36	3.33	1.17	100.62	3.62	97.69	2.31	97.59	2.41
Kae-3-*O*-Glc	16.05 ± 0.34	2.12	0.70	102.22	2.17	98.32	1.68	98.01	1.99
Iso-3-*O*-Glc	4.09 ± 0.17	4.16	1.68	100.49	2.49	96.82	3.18	95.60	4.40

Myr: myricetin; Myr-3-*O*-Glc: Myr-3-*O*-glucoside; Que: quercetin; rutin: Quer-3-*O*-Rut (with rut: rutinoside); Quer-3-*O*-Glu: Quer-3-*O*-glucuronic acid; Kae: kaempferol; Kae-3-*O*-Glc: Kae-3-*O*-glucoside; Kae-3-*O*-Rob: Kae-3-*O*-robinobioside; Kae-3-*O*-Glu: Kae-3-*O*-glucuronic acid; Iso: isorhamnetin; Iso-3-*O*-Glc: Iso-3-*O*-glucoside; RSD: relative standard deviation; HortRat: Horwitz ratio.

**Table 4:** Comparison of in vitro and cellular antioxidant capacity and in vitro skin aging enzyme inhibition of *N. nucifera* stamen vs. whole flower ethanolic extract.

Activity	Stamen Extract	Whole Flower Extract	Statistical Significance
DPPH ^1^	183.69 ± 4.84	89.91 ± 2.64	***
ABTS ^1^	60.45 ± 2.80	25.14 ± 1.35	***
ORAC ^1^	57.95 ± 2.67	37.85 ± 2.96	**
FRAP ^1^	319.60 ± 13.14	118.83 ± 3.54	***
CUPRAC ^1^	287.96 ± 4.90	102.85 ± 5.23	***
CAA ^2^	83.84 ± 7.77	57.78 ± 8.03	**
Tyrosinase ^3^	64.77 ± 6.07	47.59 ± 8.43	**
Collagenase ^3^	53.16 ± 10.27	42.88 ± 8.14	*
Elastase ^3^	25.19 ± 5.86	26.44 ± 6.14	ns
Hyaluronidase ^3^	27.21 ± 9.66	24.29 ± 5.50	ns

^1^ Antioxidant capacity expressed in µmol TEAC (Trolox-equivalent antioxidant activity); ^2^ CAA: cellular antioxidant activity expressed in % of inhibition of ROS and RNS (reactive nitrogen species) formation in yeast cells submitted to UV-induced oxidative stress; ^3^ expressed in % of enzyme inhibition; DPPH: 2,2-diphenyl-1-picrylhydrazyl; ABTS: 2,2-azinobis (3-ethylbenzthiazoline-6-sulphonic acid; ORAC: oxygen radical absorbance capacity; FRAP: ferric-reducing antioxidant power; CUPRAC: cupric ion-reducing antioxidant capacity. 1,10-Phenantroline (100 µM) was used as the specific inhibitor of collagenase leading to an inhibition of 33.6 ± 2.2%. Kojic acid (10 µM) was used as the specific inhibitor of tyrosinase leading to an inhibition of 51.2 ± 0.9%. Oleanolic acid (10 µM) was used as the specific inhibitor of elastase and hyaluronidase leading to an inhibition of 47.8 ± 1.4% and 33.5 ± 2.8%, respectively; *** significance at *p* < 0.001; ** significance at *p* < 0.01; * significance at *p* < 0.05; ns: not significant.

## Data Availability

All the data supporting the findings of this study are included in this article.

## Supplementary Materials

The following supporting information, Table S1: title; Quantification of the major flavonoids from *N. nucifera* stamen extract compared to the whole flower extract.

## Sample Availability

Samples of the compounds are available from the authors.
