# Supplementary material for: Validation of a High-Performance Liquid Chromatography with Photodiode Array Detection Method for the Separation and Quantification of Antioxidant and Skin Anti-Aging Flavonoids from Nelumbo nucifera Gaertn. Stamen Extract
Source: Molecules. 2022 Feb 7;27(3):1102. doi: 10.3390/molecules27031102 (PMC8838782; doi:10.3390/molecules27031102)
Supplement: Supplementary file 1 [file molecules-27-01102-s001.zip › molecules-1555220-supplementary.pdf]

Supplementary Materials

Validation of a High-Performance Liquid Chromatography with Photodiode Array Detection Method for the Separation and Quantification of Antioxidant and Skin Anti-Aging Flavonoids from *Nelumbo nucifera* Gaertn. Stamen Extract

Duangjai Tungmunnithum [1,2,3,*], Samantha Drouet [2] and Christophe Hano [2,3,*]

1 Department of Pharmaceutical Botany, Faculty of Pharmacy, Mahidol University, Bangkok 10400, Thailand
2 Laboratoire de Biologie des Ligneux et des Grandes Cultures, INRAE USC1328, Campus Eure et Loir,
Orleans University, 28000 Chartres, France; samantha.drouet@univ-orleans.fr
3 Le Studium Institue for Advanced Studies, 1 Rue Dupanloup, 45000 Orléans, France
* Correspondence: duangjai.tun@mahidol.ac.th (D.T.); hano@univ-orleans.fr (C.H.)

Table S1. Quantification of the major flavonoids from *N. nuficera* stamen extract compared to the whole flower extract.

	STAMEN	WHOLE FLOWER
Myr-3-Glc	5.94 ± 0.23	1.11 ± 0.90
Rut	4.68 ± 0.14	0.98 ± 0.11
Que-3-Glu	3.21 ± 0.09	1.07 ± 0.07
Kae-3-Rob	8.99 ± 0.21	2.15 ± 0.10
Kae-3-Glc	10.81 ± 0.36	3.43 ± 0.06
Kae-3-Glu	16.05 ± 0.34	4.22 ± 0.14
Iso-3-Glc	4.09 ± 0.17	1.21 ± 0.05

Mean ± standard deviations (n = 3). Myr: myricetin; Myr-3-O-Glc: Myr-3-O-glucoside; Que: quercetin; rut : rutinoside; rutin: Quer-3-O-Rut; Quer-3-O-Glu: Quer-3-O-glucuronic acid; Kae: kaempferol; Kae-3-O-Glc: Kae-3-O-glucoside; Kae-3-O-Rob: Kae-3-O-robinobioside; Kae-3-O-Glu: Kae-3-O-glucuronic acid; Iso: isorhamnetin; Iso-3-O-Glc: Iso-3-O-glucoside.
